# Allostatic load and chronic pain: a prospective finding from the national survey of midlife development in the United States, 2004–2014

**DOI:** 10.1186/s12889-024-17888-1

**Published:** 2024-02-09

**Authors:** Yunlong Liang, Cara Booker

**Affiliations:** Institute for Social and Economic Research, University of Essex, Wivenhoe Park, Colchester, Essex CO4 3SQ UK

**Keywords:** Chronic pain, Allostatic load, Biomarkers, Prospective association

## Abstract

**Background:**

Previous research has demonstrated a correlation between chronic stress and chronic pain (CP). However, there have been few studies examining the prospective association of allostatic load (AL)—the biological processes related to stress—with CP.

**Methods:**

We firstly conducted latent class analysis to identify phenotypes of AL using a community-dwelling sample, the Midlife in the United States. Multinomial logistic regression models were used to examine the prospective association between phenotypes of AL at MIDUS 2 biomarker project and the presence of CP, CP interference and the number of CP sites at MIDUS 3.

**Results:**

Three phenotypes of AL, low biological dysregulation, parasympathetic dysregulation and metabolic dysregulation, were identified. Compared to low biological dysregulation group, participants experiencing metabolic dysregulation phenotype of AL at MIDUS 2 had higher risks of having high-interference CP (RRR = 2.00, 95% CI: 1.06, 3.79, *P* < 0.05) and 3 or more CP sites (RRR = 2.03, 95% CI: 1.08, 3.83, *P* < 0.05) at MIDUS 3.

**Conclusion:**

The findings indicate that focusing on mitigating the metabolic dysfunction phenotype of AL has the potential to be an efficacious strategy for alleviating future CP bodily widespreadness and high CP interference.

**Supplementary Information:**

The online version contains supplementary material available at 10.1186/s12889-024-17888-1.

## Introduction

Chronic pain (CP) is pain that lasts or recurs for more than 3 months [1]. CP is becoming a major health issue worldwide. In the US, an estimated 20.5% of adults suffer from CP each year, causing significant burden to the healthcare system and costing over $296 billion in lost productivity [2]. The pathological progression of CP has been linked to chronic stress-related physiological dysregulation across multiple systems [3–5]. Such dysregulation has been well described by the framework of allostatic load (AL). AL is defined as the physiological ‘wear and tear’ resulting from repeated adaptations to chronic stressors [6]. Long-term response to chronic stress leads to prolonged activation of the hypothalamus-pituitary-adrenal (HPA) axis and sympathetic nervous system, resulting in elevated levels of glucocorticoids and catecholamines [7, 8]. Over time, over-accumulation of these substances can have downstream consequences and contribute to subclinical conditions across cardiovascular, metabolic, and immune systems.

In the past few decades, there has been substantial evidence indicating the association between AL and various chronic diseases and symptoms [9], however, the examination of the association between AL and CP is still in its preliminary stage. CP is closely associated with chronic stress and may involve abnormalities in several biological systems. Notably, CP patients commonly present dysregulations in the HPA axis, the autonomic nervous system, and the immune system [5, 10]. Furthermore, CP patients often exhibit a range of maladaptive stress responses, including an inability to habituate to repeated similar stressors, a failure to turn off stress responses, and altered or inefficient responses to stress [3, 7]. These dysregulations significantly align with the conditions of the AL. Therefore, some scholars suggest that CP may represent an AL disease [3].

Mixed results regarding the association between AL and CP were found among clinical samples. Research indicates that pediatric patients with pain exhibit a greater risk of experiencing AL, and AL is associated with pain-related functional impairments [11]. A prospective association between AL and CP has been suggested. A one-year longitudinal study reported a mild correlation between the AL index and pain severity among chronic low back pain patients [12]. Specifically, a set of biomarkers encompassing norepinephrine, interleukin-6, triglycerides, waist-to-hip ratio, and resting pulse rate, that demonstrated significant predictive value for chronic low back pain. However, another 6-year longitudinal study reported no association between stress response systems and chronic widespread pain (CWP) improvement [13]. While the use of validated CP assessments helped to control measurement errors, the paradoxical results may be due to inconsistencies in operationalizing chronic stress response dysregulation and in measuring CP outcomes. Additionally, the clinical samples limits the applicability of these findings to the general population.

Several population-based studies have consistently demonstrated a positive association between AL and CP in cross-sectional analyses. For example, higher levels of AL are correlated with an increased likelihood of reporting CP, especially widespread bodily pain, among adults in the U.S [14]. However, this study only computed AL based on metabolic, inflammatory, and cardiovascular biomarkers, disregarding primary mediators such as biomarkers in the HPA axis and in sympathetic nervous system [7]. Among a sample of adults over the age of 50 in England, severe CP has been associated with a high level of AL, which encompassed HPA axis biomarkers, after adjusting for sociodemographic factors, health behaviors, and chronic conditions [15]. However, the measurement of CP duration was vague, using the term ‘often’ without specific time frames. Furthermore, the cross-sectional nature limits the ability to establish causal direction between AL and CP or to account for baseline confounders that might influence CP. Additionally, the AL index in previous research primarily relied on a summative score. This computation lacks the ability to discern AL differences within each biological system or across systems [16].

Our study aimed to investigate the prospective relationship between AL and CP using a community-dwelling sample. We utilized latent class analysis (LCA) to capture the nuances of AL phenotypes [17, 18]. Additionally, we used CP measures that adheres to the definition of CP in terms of pain duration [19], thereby enhancing the validity of our pain assessments. Our examination was also adjusted for a range of factors including sociodemographic characteristics, health-related behaviors, multiple chronic conditions, and detailed medication information. We hypothesized that AL phenotypes would be prospectively associated with increased risk of experiencing CP, increased number of pain locations, and greater pain interference after seven years.

## Methods

### Data

This study used the Midlife in the United States (MIDUS) from 2004 to 2014, including two main survey waves (MIDUS 2 and MIDUS 3) and a Biomarker Project of MIDUS 2. MIDUS is a national longitudinal study focusing on individual social status, psychological profiles, and biological processes of aging, initiated between 1995 and 1996 and followed 7,108 non-institutionalized Americans aged 25 to 74 in the contiguous United States. The main survey collected data by phone interviews and self-administered questionnaires.

**Fig. 1:**
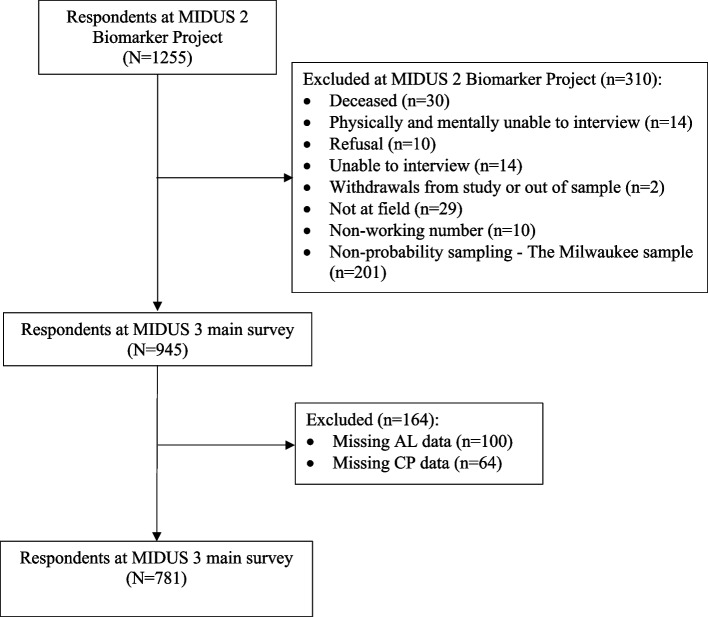
Flow diagram for the study cohort

 Of the participants, 1,255 were involved in the Biomarker Project of MIDUS 2, conducted from 2004 to 2009. Samples meeting the following criteria were incorporated into the analyses (see Fig. 1): (1) samples that participated in the biomarker program and the MIDUS 3 follow-up survey, (2) samples that provided complete information on the major variables (AL and CP). The MIDUS is publicly accessible secondary data. More details of the study are available on the MIDUS website (Available at: http://midus.wisc.edu/).

### Measures

#### Allostatic load

AL biomarkers were collected from the Biomarker Project of MIDUS 2. The project collected 12-hour urine samples, fasting blood samples, as well as nervous system function data from respondents during a one-day stay at a General Clinical Research Center (GCRC) of either UCLA, University of Wisconsin, or Georgetown University, depending on the residence of respondents [20].

Following previous studies [7, 16, 21], AL was constructed into seven physiological systems from 27 biomarkers (shown in Table 1). A high-risk quartile of biomarkers were used [22]. Dehydroepiandrosterone sulfate (DHEA-S) and cortisol in the upper or lower 25th quartile were regarded as at high risk. When high-frequency heart rate variability (HFHRV), low-frequency heart rate variability (LFHRV), root mean square of successive differences (RMSSD), standard deviation of heart beat to heart beat intervals (SDRR), and high-density lipoprotein (HDL) cholesterol strength fell within their lower 25th quartile ranges, individuals were classified as high risk. Other biomarkers falling into their upper 25th quartile were assigned to the high-risk range. Then, biomarkers in their high-risk quartile were coded as 1; otherwise, 0. The high-risk thresholds are detailed in Table 1.

**Table 1:** Values for high-risk quartiles

Biomarkers	Simple High Risk Quartile
**Hypothalamic Pituitary Adrenal Axis**	
DHEA-s (ug/dL)	≤ 51 or ≥ 141
Urine cortisol (µg/g)	≤ 6.7 or ≥ 19
**Sympathetic Nervous System**	
Urine epinephrine (µg/g)	≥ 2.464
Urine norepinephrine (µg/g)	≥ 32.964
Urine Dopamine (µg/g)	≥ 182.964
**Parasympathetic Nervous System**	
HFHRV	≤ 55.9
LFHRV	≤ 103.4
RMSSD	≤ 12.02
SDRR (ms)	≤ 23.27
**Cardiovascular**	
Resting heart rate (bpm)	≥ 79.8
Resting systolic blood pressure (SBP) (mmHg)	≥ 144
Resting diastolic blood pressure (mmHg)	≥ 82
**Metabolic-glucose**	
Fasting glucose	≥ 105
Hemoglobin A1c (HbA1c) (%)	≥ 6.242
Homeostasis model of insulin resistance (HOMA-IR)	≥ 4.36
**Metabolic-lipids**	
Triglycerides (mg/dL)	≥ 156
Waist-to-hip ratio (WHR)	≥ 0.965
Body mass index (BMI) (kg/m^2^)	≥ 33.028
Low-density lipoprotein (LDL) cholesterol (mg/dL)	≥ 127
High-density lipoprotein (HDL) cholesterol (mg/dL)	≤ 43
**Inflammation**	
C-reactive protein (CRP) (mg/L)	≥ 3.655
Interleukin-6 (IL6) (pg/mL)	≥ 1.23
Tumor necrosis factor-α (TNF-α) (pg/mL)	≥ 2.51
Fibrinogen (mg/dL)	≥ 399
Soluble endothelial leukocyte adhesion molecule-1 (sE-Selectin) (ng/mL)	≥ 51.88
Soluble intercellular adhesion molecule-1 (ICAM-1) (ng/mL)	≥ 335.185
Blood fasting insulin-like growth factor 1 (IGF1) (ng/mL)	≥ 157

Then, LCA was used to capture the phenotypes of AL (package “poLCA” in R). The binary biomarkers were fitted into 1–7 clusters, and the selection of the optimum number of cluster was based on log-likelihood, Akaike Information Criterion (AIC), Bayesian Information Criterion (BIC), entropy, and interpretability of classification. Regarding entropy, an ideal value is close to 1, and above 0.8 is acceptable [23]. As for AIC and BIC, lower values indicate a better fit [24]. However, BIC tends to favor simpler models in larger samples due to its complexity penalty, while AIC may lean towards more complex models. Given these considerations, seeking points of inflection or plateauing for BIC and AIC can balance model complexity against the risk of overfitting [24]. Also, the classification should be meaningful from a clinical or a biological perspective [24]. Additionally, each cluster should have at least 10% of the sample [23, 24]. 5000 iterations were set to generate convergent estimation for each LCA model.

#### Outcome: chronic pain

CP interference and the number of CP sites from MIDUS 3 were utilized. Respondents were first asked “Do you have chronic pain, that is do you have pain that persists beyond the time of normal healing and has lasted from anywhere from a few months to many years?” An affirmative response indicated the presence of CP and the respondents were then asked about CP interference. A pain interference index was generated by calculating a mean score of how much pain interfered with respondents’ activity, mood, relations, sleep, and enjoyment, ranging from 0 to 10 [25, 26]. Then, the pain interference index was further categorized into no pain, low interference pain (≤ 4), and high interference pain (> 4) as categorical variable [25]. In addition, if respondents reported having CP, they were asked about the location of the pain, including head, neck, back, arms, legs, shoulders, hips, knees, and other sites. We summed up the pain sites into an index and then categorized it into no pain, 0–2 sites, or 3 or more sites as a categorical variable [26, 27].

#### Covariates

Covariates were selected by current knowledge about the association between AL and CP [14, 15, 28]. Sociodemographic covariates were obtained from the MIDUS 2 main survey and were coded as categorical variables except for the age variable, which was treated as continuous. Sociodemographic covariates included gender (ref: males), age, ethnicity (ref: White), educational attainment (i.e., the highest educational certificate a respondent had obtained, ref: high school or less), marital status (ref: Married), and the income-to-needs ratio (INR, ref: Affluent) [29] which was computed by dividing total household income by Federal Poverty Threshold [30]. Additionally, behavior factors from the MIDUS 2 Biomarker Project were considered. They were alcohol intake status (ref: Moderate + drinker), smoking status (ref: Current smokers), and categories of the metabolic equivalent of task (MET, ref: Between 500 and 1000 min per week) minutes per week [26, 31]. Also, the time gap between the two data collections was controlled for. Finally, adverse childhood experiences (ACEs) also possibly confound the relationship between AL and CP [32, 33]. In this case, we considered emotional abuse and physical abuse from parents. The ACE data were retrospectively collected in the MIDUS 1 and were treated as ordinal variables.

Multimorbidity was also adjusted for [28, 34]. The chronic condition index summed up a count of “Yes” responses to the chronic conditions-related questions [20]. Then, the index was coded as a binary variable (Ref: <2) and the index more than 2 was regarded as multimorbidity. Since mental health conditions were already incorporated in this variable, there were no extra adjustments for depression and anxiety.

MIDUS 2 Biomarker Project enhanced medication reports by linking medication names and IDs to Generic Names and Lexi-Data database and asking respondents for their reasons for taking medications [20]. A binary variable was created to represent whether a participant had taken any medication from a selection of antihyperlipidemic agents, beta adrenergic blocking agents, antihypertensive combinations, anxiolytics sedatives and hypnotics, antidiabetic agents, sex hormones, thyroid hormones, antidepressants, and analgesics, including opioids and non-opioids.

### Analysis

#### Statistical methods

Regression models were chosen according to types of CP variables. For a binary CP variable, logistic regressions were used. The number of pain location and pain interference were categorical variables, therefore, multinomial logistic regressions were utilized. All main analyses presented were fully adjusted for relevant confounders to reduce spurious associations and were generated from the complete cases.

Three sensitivity analyses were applied. Firstly, data missingness can lead to biased estimation [35, 36]. Multiple imputation (MI) using the R package “MICE” [37] was employed to address item nonresponse, based on the assumption of missing at random (MAR). Missing covariates were imputed in accordance with the specific distribution of each item, as recommended [36]. Twenty imputed datasets were generated, and the coefficients from all statistical models were combined using Rubin’s rules. ANOVA tests and chi-squared tests were performed respectively for continuous variables and categorical variables to check the similarity of imputed datasets and the observed dataset. Secondly, bootstrapping method was used to estimate the variability and robustness of coefficients [38]. A total of 5000 bootstrap samples were generated with replacement, each with the same sample size as the original dataset. The bootstrapping process was conducted by R. Finally, CP status at MIDUS 2 was incorporated into the model and the binary measure of medication intake at MIDUS 2 was substituted with specific individual medications.

## Results

### Descriptive statistics

Table 2 displays the descriptive statistics of the analytic sample (*N* = 781). 62.7% of participants reported no CP and 37.3% of participants reported the presence of CP. 24.6% of participants had low-interference pain, and 12.7% of participants had high-interference pain. In terms of the number of pain locations, 23.8% of participants reported 0–2 pain sites and 13.4% of participants reported 3 or more pain sites. The majority of respondents were females, non-Hispanic whites, affluent, and married, with over 48% of respondents being highly educated (above high school degree). Additionally, there were no significant differences between observed dataset and imputed datasets, supporting the validity of the imputation process.

**Table 2:** Sample description

	Observed dataset				Imputed dataset			
Variable	Mean / N	SD / Proportion	Median	Proportion of available value	Mean / N	SD / Proportion	Median	Test
Pain interference at MIDUS 3	781			1.000				X^2^ = 0
No pain	490	62.74%				62.74%		
Low interference pain	192	24.58%				24.58%		
High interference pain	99	12.68%				12.68%		
Number of pain sites at MIDUS 3	781			1.000				X^2^ = 0
No pain	490	62.74%				62.74%		
0–2	186	23.81%				23.81%		
3+	105	13.44%				13.44%		
Allostatic load phenotypes	781			1.000				X^2^ = 0
Baseline	403	51.60%				51.60%		
Parasympathetic dysregulation	189	24.20%				24.20%		
Metabolic dysregulation	189	24.20%				24.20%		
**Sociodemographic**								
Education	780			0.999				X^2^ = 0
High school or less	397	50.90%				50.90%		
Bachelor’s degree	233	29.90%				29.90%		
Master’s degree and above	150	19.20%				19.20%		
Gender	781			1.000				X^2^ = 0
Male	351	44.90%				44.90%		
Female	430	55.10%				55.10%		
Age	54	10.907	54	1.000	54	10.9	54	F = 0
Race/ethnicity	780			0.999				X^2^ = 0
White	723	92.70%				92.70%		
Non-white	57	7.30%				7.30%		
Marital Status	780			0.999				X^2^ = 0
Married	570	73.10%				73.10%		
Divorced & Separated	113	14.50%				14.50%		
Never married & Widowed	97	12.40%				12.40%		
Income-to-needs ratio	767			0.982				X^2^ = 0.008
Affluent	437	57%				57%		
Adequate-income	211	27.50%				27.50%		
Low-income or below	119	15.50%				15.50%		
**Year gap between data collections**								
MIDUS 2 Biomarker Project to MIDUS 3	6.7	1.249	6.833	1.000	6.7	1.249	6.833	F = 0
**Childhood adversity**								
Childhood parent emotional abuse	724			0.927				X^2^ = 0.1
1 (Never)	225	31.10%				30.70%		
1.5	111	15.30%				15.40%		
2	200	27.60%				27.40%		
2.5	101	14%				14.20%		
3 (Most frequent)	87	12%				12.20%		
Childhood parent physical abuse	732			0.937				X2 = 0.147
1 (Never)	309	42.20%				41.90%		
1.5	116	15.80%				16.20%		
2	184	25.10%				24.90%		
2.5	71	9.70%				10%		
3 (Most frequent)	52	7.10%				7.10%		
**Health behavior**								
Total number of Metabolic Equivalent of Task (MET) minutes per week	776			0.994				X^2^ = 0.001
500–1000	151	19.50%				19.50%		
Greater than 1000	319	41.10%				41.10%		
Less than 500	306	39.40%				39.40%		
Smoking behavior	780			0.999				X^2^ = 0
Current Smoker	87	11.20%				11.10%		
Ex-Smoker	247	31.70%				31.70%		
Non-Smoker	446	57.20%				57.20%		
Drinking behavior	781			1.000				X^2^ = 0
Moderate + drinker	308	39.40%				39.40%		
Light drinker	228	29.20%				29.20%		
Non-drinker or rarely drink	245	31.40%				31.40%		
**Health conditions**								
Multimorbidity	781			1.000				X^2^ = 0
<2	168	21.50%				21.50%		
2+	613	78.50%				78.50%		
**Medication**								
Medication intake	781			1.000				X^2^ = 0
Yes	204	26.10%				26.10%		
No	577	73.90%				73.90%		

Statistical significance markers: * *p* < 0.1; ** *p* < 0.05; *** *p* < 0.01

Supplement Table 1 presents the fit statistics for latent class model with 1–7 clusters, the 3-cluster model was considered the optimal clustering. Despite the continuous reduction in AIC and BIC, along with the progressive improvement in log-likelihood, the enhancement in the fitness of the model with 4 and 5 clusters was rather moderate. On the other hand, the 3-cluster model exhibited the best entropy, suggesting a good classification. Additionally, the 3-cluster model had an ample number of observations within each cluster and presented meaningful separation. Therefore, the 3-cluster model was adopted.

**Fig. 2:**
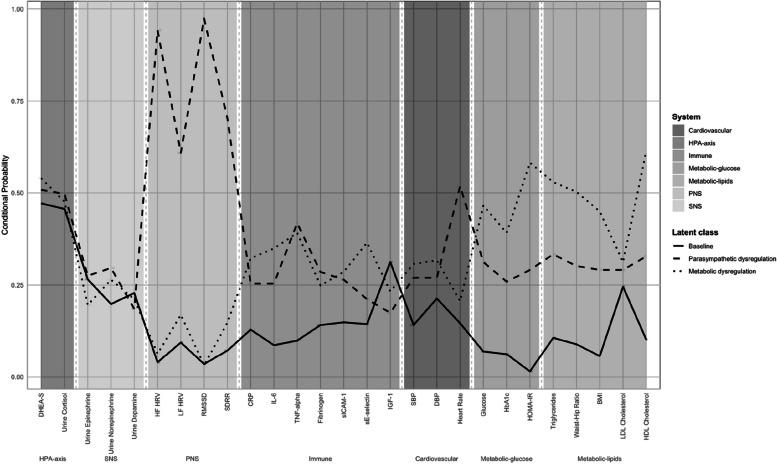
Identified phenotypes of allostatic load

According to Supplement Table 2, class 1 is designated as ‘Baseline’ due to its association with a low risk across most biomarkers. Class 2, termed ‘Parasympathetic Dysregulation,’ is distinguished by significantly lower values in HFHRV, LFHRV, RMSSD, and SDRR, suggesting potential impairments in parasympathetic system functioning. Class 3 is characterized by marked increases in fasting glucose, HbA1c, HOMA-IR, triglycerides, WHR, and BMI, coupled with a notable decrease in HDL concentrations. These characteristics are consistent with the physiological patterns commonly observed in metabolic dysregulation. Figure 2 shows the phenotypes of AL. 51.6% of the participants were classified as low AL risk group, 24.2% of participants were in the phenotype of parasympathetic dysregulation, and an additional 24.2% demonstrated signs of metabolic dysregulation.

### Model results

Table 3 presents regression results. In the fully adjusted binary logistic regression models, there was no statistically significant association between any AL dysregulation phenotype and CP status compared to the low AL risk phenotype.

**Table 3:** Results from the logistic regression for the association between AL at MIDUS 2 Biomarker Project and CP status at MIDUS 3^†^

	No CP vs. reporting CP in MIDUS 3
AL phenotypes	Odds ratios (95% CI)
**Baseline**	Ref
**Parasympathetic dysregulation**	
** Main analysis**	0.97 (0.64, 1.48)
** Sensitivity analysis**	
Multiple Imputation	1.04 (0.7, 1.55)
Bootstrapping Method (5000 iterations)	0.85 (0.51, 1.43)
Adjustment for CP at MIDUS 2 and individual medications^a^	1.01 (0.64, 1.6)
**Metabolic dysregulation**	
** Main analysis**	1.18 (0.76, 1.81)
** Sensitivity analysis**	
Multiple Imputation	1.14 (0.77, 1.7)
Bootstrapping Method (5000 iterations)	1.40 (0.8, 2.45)
Adjustment for CP at MIDUS 2 and individual medications^a^	1.18 (0.74, 1.89)

The bold values denote statistically significant results

^a^Medications included antihyperlipidemic agents, beta adrenergic blocking agents, antihypertensive combinations, anxiolytics sedatives and hypnotics, sex hormones, thyroid hormones, antihistamines, antidepressants, analgesics (both opioids and non-opioids)

Statistical significance markers: * *p* < 0.05; ** *p* < 0.01; *** *p* < 0.001  (No *, **, *** in the table indicates no statistical signficance)

†Adjusted for gender, age at MIDUS 2, race/ethnicity, marital status at MIDUS 2, INR at MIDUS 2, emotional/physical abuse from parents, multimorbidity at MIDUS 2 Biomarker Project, MET, drinking behavior, smoking behavior, medication intake (yes/no) and year gap between MIDUS 2 Biomarker Project and MIDUS 3 main surveys

In the multinomial logistic regression models (Table 4), the prospective association between the metabolic dysregulation phenotype and high-interference CP was significant (RRR = 2.00, 95% CI: 1.06, 3.79, *P* < 0.05), compared to the baseline phenotype. In the prospective association between the number of pain sites and biological dysregulation phenotypes, metabolic dysregulation was significantly associated with 3 or more CP sites (RRR = 2.03, 95% CI: 1.08, 3.83, *P* < 0.05). There were no other significant associations found. In the sensitivity analyses, the results remained similar. The similar results generated from the imputed datasets indicated that data missingness did not significantly biased the estimates. Also, the results generated from the bootstrapping samples were similar to the main analyses, indicating that the association was expected to persist even when accounting for potential uncertainties. Finally, after extra adjusting for medication intakes as separate factors and CP status at MIDUS 2, the results remained stable. Supplement Table 3 displays the full models for examining the relationship between AL phenotypes and CP status, CP interference, and the number of CP locations.

**Table 4:** Results from the multinomial logistic regression for the association between AL at MIDUS 2 Biomarker Project and CP interference and the number of CP sites at MIDUS 3^†^

	**No pain vs. low-interference pain**	**No pain vs. high-interference pain**
**AL phenotypes**	**Relative risk ratios (95% CI)**	**Relative risk ratios (95% CI)**
**Baseline**	Ref	Ref
**Parasympathetic dysregulation**		
** Main analysis**	0.87 (0.54, 1.39)	1.24 (0.65, 2.39)
** Sensitivity analysis**		
Multiple Imputation	0.96 (0.61, 1.49)	1.22 (0.66, 2.26)
Bootstrapping Method (5000 iterations)	0.82 (0.49, 1.38)	0.99 (0.41, 2.38)
Adjustment for CP at MIDUS 2 and individual medications^a^	0.93 (0.56, 1.53)	1.23 (0.60, 2.55)
**Metabolic dysregulation**		
** Main analysis**	0.92 (0.56, 1.52)	2.00 (1.06, 3.79)*
** Sensitivity analysis**		
Multiple Imputation	0.92 (0.58, 1.46)	1.82 (1.01, 3.28)*
Bootstrapping Method (5000 iterations)	1.08 (0.58, 2.02)	2.46 (1.10, 5.47)*
Adjustment for CP at MIDUS 2 and individual medications^a^	0.94 (0.55, 1.59)	2.03 (1.01, 4.11)*
	**No pain vs. 0–2 pain locations**	**No pain vs. 3 + pain locations**
**AL phenotypes**	**Relative risk ratios (95% CI)**	**Relative risk ratios (95% CI)**
**Baseline**	Ref	Ref
**Parasympathetic dysregulation**		
** Main analysis**	0.84 (0.51, 1.36)	1.30 (0.69, 2.44)
** Sensitivity analysis**		
Multiple Imputation	0.91 (0.58, 1.45)	1.33 (0.73, 2.39)
Bootstrapping Method (5000 iterations)	0.85 (0.50, 1.46)	0.83 (0.27, 2.62)
Adjustment for CP at MIDUS 2 and individual medications^a^	0.90 (0.54, 1.51)	1.22 (0.61, 2.42)
**Metabolic dysregulation**		
** Main analysis**	0.89 (0.54, 1.47)	2.03 (1.08, 3.83)*
** Sensitivity analysis**		
Multiple Imputation	0.91 (0.57, 1.44)	1.85 (1.03, 3.34)*
Bootstrapping Method (5000 iterations)	1.00 (0.55, 1.81)	2.57 (1.15, 5.76)*
Adjustment for CP at MIDUS 2 and individual medications^a^	0.89 (0.52, 1.52)	2.09 (1.06, 4.11)*

The proportional odds assumption for ordinal logistic regression was violated. Therefore, multinomial logistic regression was opted for

^a^Medications included antihyperlipidemic agents, beta adrenergic blocking agents, antihypertensive combinations, anxiolytics sedatives and hypnotics, sex hormones, thyroid hormones, antihistamines, antidepressants, analgesics (both opioids and non-opioids)

†Adjusted for gender, age at MIDUS 2, race/ethnicity, marital status at MIDUS 2, INR at MIDUS 2, emotional/physical abuse from parents, multimorbidity at MIDUS 2 Biomarker Project, MET, drinking behavior, smoking behavior, medication intake (yes/no) and year gap between MIDUS 2 Biomarker Project and MIDUS 3 main surveys

Statistical significance markers: * *p* < 0.05; ** *p* < 0.01; *** *p* < 0.001 (no **, *** in the table indicates no such statistical significance was found); the bold values denote statistically significant results

### Predicted probabilities

Table 5 presents the adjusted prevalence for CP outcomes grouped by AL phenotypes. Using the average adjusted predicted probabilities from the models, we calculated the probability of CP outcomes by AL phenotypes. The metabolic dysregulation phenotype was significantly associated with high-interference pain and 3 or more CP sites as shown in Table 4. Respondents with the metabolic dysregulation phenotype were more likely to experience a higher degree of CP conditions than those with a low AL risk profile. Specifically, those with metabolic dysregulation driven AL had a 4.88% adjusted probability of reporting high pain interference and had a 4.58% adjusted probability of reporting more than 3 pain locations. In contrast, these probabilities were lower, at 2.48% and 2.29% respectively, among respondents with a baseline AL profile.

**Table 5:** Adjusted prevalence for CP outcomes grouped by AL phenotypes

**CP status**	**No pain**	**Reporting CP**	
**AL phenotypes**	**Average adjusted predicted probabilities**		
Baseline	89.02%	10.98%	
Parasympathetic dysregulation	89.31%	10.69%	
Metabolic dysregulation	87.34%	12.66%	
**CP interference**	**No pain**	**Low interference pain**	**High interference pain**
**AL phenotypes**	**Average adjusted predicted probabilities**		
Baseline	89.74%	7.77%	2.48%
Parasympathetic dysregulation	90.14%	6.76%	3.10%
Metabolic dysregulation	88.11%	7.02%	**4.88%**
**The number of CP locations**	**No pain**	**0–2**	**3+**
**AL phenotypes**	**Average adjusted predicted probabilities**		
Baseline	90.17%	7.54%	2.29%
Parasympathetic dysregulation	90.66%	6.35%	3.00%
Metabolic dysregulation	88.80%	6.62%	**4.58%**

Findings in bold are statistically significant at *p* < 0.05 based on binary/multinomial logistic regression results

## Discussion

The present study identified three phenotypes of AL through LCA, encompassing low levels of biological dysregulation, AL driven by parasympathetic dysregulation, and AL driven by metabolic dysregulation. Also, consistent with previous research [14, 15, 39, 40], we found that AL driven by metabolic dysregulation is associated with more severe CP interference and a greater number of CP sites. For instance, a cross-sectional study based on a sample of population aged over 50 in the UK revealed that, after controlling for sociodemographic factors and comorbid conditions, high-risk biomarker, defined by the upper quartile and including HDL, HBA1c, and WHR, are related to increased severity of CP [15]. Similarly, in American adults, higher BMI and triglyceride levels are associated with a higher prevalence of widespread bodily pain [14].

Compared to previous studies, our research offers several advantages. Firstly, we employed a more comprehensive set of biomarkers, including those from the HPA axis, and the sympathetic and parasympathetic nervous systems, to construct a more valid AL measurement [7]. Moreover, our use of LCA to identify AL phenotypes captured the common variability of biomarkers, while previous studies that used single biomarkers for regression with CP to examine the AL driving systems of CP overlooked the interrelationships among biomarkers within the AL framework [14, 15]. On the other hand, prior operationalizations of AL, based on summative computation that assigns equal weight to each biomarker, may obscure the specific impacts of different AL components on CP. In summary, LCA offers a nuanced method for exploring the specific components of AL that drive CP.

Furthermore, this study’s strengths include its prospective design, community-dwelling sample, adjustments for early confounders, and the substantial avoidance of trivial and recent pain in measurement by adhering to the definition of CP in terms of pain duration. Thus far, this research may be the first community-dwelling study to examine the prospective association between AL and CP.

However, this study also has limitations. Firstly, the measurement of pain is self-reported. Even when controlling for potential reporting biases from relevant sociodemographic factors, unobserved factors can still introduce biases in pain assessment. Furthermore, the variability in CP measures across various surveys partly limits the comparability of findings. For instance, the MIDUS survey assesses pain interference, which differs from the pain severity measurements used in other studies. While pain interference is associated with pain severity, the association is affected by patients’ beliefs about pain, their tendency towards catastrophizing, and their pain coping strategies. These factors can alter the relationship between pain interference and pain severity [41]. Therefore, there is a need for further prospective research to explore the link between AL and CP severity in more depth.

Additionally, the available data on AL was only collected in MIDUS 2 during our research, however, the upcoming biomarker data present opportunities for future research on the association between AL trajectories and the development of CP. Also, the sample composition is predominantly white people, and future studies focusing on ethnic minorities are encouraged. Moreover, our findings from the U.S. data may not generalize to other countries due to differences in health care systems, lifestyle choices, and the impact of sociocultural variables on the reporting and perception of pain. Lastly, this study only examined the prospective association in one direction and future research on the reverse association may be beneficial elucidate the causal direction.

While the underlying mechanism remains undetermined, several potential explanations could account for the prospective positive association between the metabolic dysregulation phenotype of allostatic load and both high-interference pain as well as an increased number of pain sites. The AL model proposes, when undergoing repeated stress adaptation, the prolonged secretion of stress hormones and inflammatory cytokines can disrupt the normal regulation of downstream physiological systems, such as the metabolic system [7]. Dyslipidemia and high BMI may be associated with upregulation of cytokines, leading to low-grade inflammation, a condition frequently observed in patients with fibromyalgia [42]. Additionally, a high waist-to-hip ratio may be related to structural changes in intervertebral discs and being consistently subjected to high biomechanical loads [43]. This highlights the significant role that metabolic dysregulation related to adiposity may play in low back pain. Meanwhile, elevated blood glucose is associated with peripheral neuropathy or synergistically interacts with high BMI and the sequential inflammation, thereby potentially increasing the likelihood of experiencing daily pain [44]. Also, metabolic dysregulation could potentially reduce the pain activation threshold via its interplay with inflammatory mechanisms. This interaction may intensify pain response by increasing synaptic strength and reducing inhibition, allowing even low-threshold stimuli to activate pain pathways [45, 46].

Nevertheless, we did not find any prospective associations between AL driven by the parasympathetic nervous system and CP. Low parasympathetic nervous system activity may represent low capacity to respond to chronic stress. A meta-analysis, which thoughtfully sieved through 26 moderate-high-quality studies from a pool of 17,350 publications, uncovered that biomarkers relating to the parasympathetic nervous system (LFHRV, HFHRV, RMSSD, R-R interval, and SDRR) exhibited an association with CP [47]. However, the association appears to be predominantly influenced fibromyalgia and its significance may vary across CP conditions [5]. CP may also maladapt parasympathetic nervous system directly. Therefore, future research is encouraged to focus on exploring the potential links between the parasympathetic nervous system and different subtypes of CP to clarify these relationships.

## Conclusion

In conclusion, our findings indicate that metabolic dysregulation as a phenotype of AL is prospectively associated with high-interference CP and 3 or more CP sites. Differentiating nuances of biological dysregulation of AL could facilitate the development of precise clinical interventions aimed at specific biological mechanisms, which may alleviate the impacts of AL on the conditions of CP.

## Supplementary Information

**Additional file 1.**

**Additional file 2.**

**Additional file 3.**

## Data Availability

MIDUS data are freely available to the public via the Web by opening an Inter-university Consortium for Political and Social Research user account.
