# Correction to: Allostatic load and chronic pain: a prospective finding from the national survey of midlife development in the United States, 2004–2014

**DOI:** 10.1186/s12889-024-20669-5

**Published:** 2024-11-19

**Authors:** Yunlong Liang, Cara Booker

**Affiliations:** Institute for Social and Economic Research, University of Essex, Wivenhoe Park, Colchester, Essex CO4 3SQ UK

**Correction to: BMC Public Health 24, 416 (2024)**

10.1186/s12889-024-17888-1

The original publication of this article contained 2 errors:

- A repetition in the last paragraph of the covariates and the footnotes of Tables 3 and 4.
- A wrong version of supplementary file 3.
  - The authors used the as.factor() function in R Studio to specify the binary pain indicator coded as 0 (no pain) and 1 (pain) as a categorical variable for regression. However, as.factor() in R Studio automatically reassigns 0 to level 2 and sets “1” (pain) as the reference group.

The incorrect and correct text are shown below, the old and new supplementary file are provided in this correction article.

These errors do not affect the other text, results, or conclusions of the paper. The original article has been updated.

**Table:** 

Incorrect	Correct
**Covariates**	
A binary variable was created to represent whether a participant had taken any medication from a selection of antihyperlipidemic agents, beta adrenergic blocking agents, antihypertensive combinations, **analgesics**, anxiolytics sedatives and hypnotics, antidiabetic agents, sex hormones, thyroid hormones, antidepressants, and analgesics, including opioids and non-opioids	A binary variable was created to represent whether a participant had taken any medication from a selection of antihyperlipidemic agents, beta adrenergic blocking agents, antihypertensive combinations, anxiolytics sedatives and hypnotics, antidiabetic agents, sex hormones, thyroid hormones, antidepressants, and analgesics, including opioids and non-opioids.
**Tables 3 and 4 footnote**	
Medications included antihyperlipidemic agents, beta adrenergic blocking agents, antihypertensive combinations, **analgesics**, anxiolytics sedatives and hypnotics, sex hormones, thyroid hormones, antihistamines, antidepressants, analgesics (both opioids and non-opioids)	Medications included antihyperlipidemic agents, beta adrenergic blocking agents, antihypertensive combinations, anxiolytics sedatives and hypnotics, sex hormones, thyroid hormones, antihistamines, antidepressants, analgesics (both opioids and non-opioids)

## Electronic supplementary material

Below is the link to the electronic supplementary material.

Supplementary Material 1: Correct supplementary file 3

Supplementary Material 2: Incorrect supplementary file 3

