# Supplementary material for: Allostatic load and chronic pain: a prospective finding from the national survey of midlife development in the United States, 2004–2014
Source: BMC Public Health. 2024 Feb 9;24:416. doi: 10.1186/s12889-024-17888-1 (PMC10854121; doi:10.1186/s12889-024-17888-1)
Supplement: Supplementary file 1 — Additional file 1. [file 12889_2024_17888_MOESM1_ESM.docx]

**SUPPLEMENT TABLE 1 Fit statistics for latent classes of allostatic load**

| **Model - Class k** | **llik (maximum value of the log-likelihood)** | **AIC** | **BIC** | **Entropy** | **Class 1 %** | **Class 2 %** | **Class 3 %** | **Class 4 %** | **Class 5 %** | **Class 6 %** | **Class 7 %** |
| --- | --- | --- | --- | --- | --- | --- | --- | --- | --- | --- | --- |
| **1** | -11670.67 | 23395.33 | 23521.17 | NaN | 1.00 |  |  |  |  |  |  |
| **2** | -11067.14 | 22244.28 | 22500.61 | 0.85 | 0.62 | 0.38 |  |  |  |  |  |
| **3** | -10813.98 | 21793.96 | 22180.79 | 0.86 | 0.24 | 0.51 | 0.25 |  |  |  |  |
| **4** | -10706.04 | 21634.09 | 22151.41 | 0.85 | 0.23 | 0.18 | 0.49 | 0.10 |  |  |  |
| **5** | -10606.58 | 21491.16 | 22138.98 | 0.84 | 0.17 | 0.14 | 0.43 | 0.11 | 0.15 |  |  |
| **6** | -10539.69 | 21413.38 | 22191.70 | NaN | 0.18 | 0.31 | 0.10 | 0.18 | 0.05 | 0.19 |  |
| **7** | -10463.27 | 21316.53 | 22225.35 | NaN | 0.11 | 0.12 | 0.17 | 0.11 | 0.25 | 0.13 | 0.12 |
