# Supplementary material for: Allostatic load and chronic pain: a prospective finding from the national survey of midlife development in the United States, 2004–2014
Source: BMC Public Health. 2024 Feb 9;24:416. doi: 10.1186/s12889-024-17888-1 (PMC10854121; doi:10.1186/s12889-024-17888-1)
Supplement: Supplementary file 2 — Additional file 2. [file 12889_2024_17888_MOESM2_ESM.docx]

**SUPPLEMENT TABLE 2 Biomarkers levels stratified by AL phenotype**

| **Allostatic load driven pattern** | **Class 1** | | **Class 2** | | **Class 3** | |  |
| --- | --- | --- | --- | --- | --- | --- | --- |
| **Biomarkers** | **Mean** | **Median** | **Mean** | **Median** | **Mean** | **Median** | **Test** |
| **Hypothalamic Pituitary Adrenal Axis** |  |  |  |  |  |  |  |
| DHEA-s (ug/dL) | 110 | 94 | 106 | 86 | 115 | 90 | F=0.688 |
| Urine cortisol (μg/g) | 17 | 15 | 15 | 11 | 13 | 10 | F=5.782^***^ |
| **Sympathetic Nervous System** |  |  |  |  |  |  |  |
| Urine epinephrine (μg/g) | 2.1 | 1.8 | 2 | 1.8 | 1.7 | 1.4 | F=6.251^***^ |
| Urine norepinephrine (μg/g) | 26 | 24 | 28 | 27 | 28 | 24 | F=2.785^*^ |
| Urine Dopamine (μg/g) | 151 | 142 | 144 | 136 | 146 | 133 | F=1.37 |
| **Parasympathetic Nervous System** |  |  |  |  |  |  |  |
| High-frequency HRV | 348 | 176 | 31 | 30 | 371 | 141 | F=15.035^***^ |
| Low-frequency HRV | 562 | 339 | 112 | 90 | 457 | 258 | F=36.218^***^ |
| RMSSD | 26 | 21 | 8.5 | 8.6 | 25 | 19 | F=88.199^***^ |
| SDRR (m s) | 41 | 36 | 21 | 20 | 37 | 34 | F=115.491^***^ |
| **Cardiovascular** |  |  |  |  |  |  |  |
| Resting SBP (mmHg) | 126 | 126 | 133 | 131 | 136 | 137 | F=30.225^***^ |
| Resting DBP (mmHg) | 74 | 73 | 76 | 76 | 77 | 77 | F=9.386^***^ |
| Resting heart rate (bpm) | 70 | 70 | 80 | 80 | 71 | 71 | F=74.174^***^ |
| **Inflammation** |  |  |  |  |  |  |  |
| CRP (mg/L) | 1.8 | 0.89 | 3.1 | 1.6 | 3.2 | 2.2 | F=13.511^***^ |
| IL6 (pg/mL) | 0.76 | 0.63 | 1.1 | 0.92 | 1.2 | 1 | F=26.438^***^ |
| TNF-α (pg/mL) | 1.9 | 1.9 | 2.5 | 2.3 | 2.4 | 2.3 | F=47.766^***^ |
| Fibrinogen (mg/dL) | 322 | 319 | 356 | 356 | 349 | 345 | F=14.897^***^ |
| ICAM-1 (ng/mL) | 264 | 244 | 297 | 276 | 297 | 289 | F=12.488^***^ |
| E-Selectin (ng/mL) | 37 | 34 | 41 | 38 | 49 | 45 | F=23.056^***^ |
| Blood Fasting IGF1 (Insulin-like Growth Factor 1) ng/mL) | 133 | 126 | 123 | 115 | 119 | 113 | F=6.492^***^ |
| **Metabolic-glucose** |  |  |  |  |  |  |  |
| Fasting glucose | 93 | 93 | 102 | 98 | 113 | 103 | F=51.566^***^ |
| Hemoglobin A1c% | 5.7 | 5.7 | 6.1 | 5.9 | 6.3 | 6 | F=37.938^***^ |
| HOMA-IR | 1.9 | 1.7 | 3.8 | 2.7 | 5.8 | 4.7 | F=107.247^***^ |
| **Metabolic-lipids** |  |  |  |  |  |  |  |
| Triglycerides (mg/dL) | 100 | 92 | 148 | 121 | 181 | 162 | F=80.591^***^ |
| WHR | 0.85 | 0.85 | 0.92 | 0.91 | 0.95 | 0.96 | F=89.071^***^ |
| BMI | 26 | 26 | 30 | 30 | 33 | 32 | F=119.981^***^ |
| LDL cholesterol (mg/dL) | 105 | 103 | 110 | 104 | 109 | 102 | F=1.358 |
| HDL cholesterol (mg/dL) | 62 | 59 | 53 | 51 | 43 | 41 | F=85.106^***^ |
