# Supplementary material for: Correction to: Allostatic load and chronic pain: a prospective finding from the national survey of midlife development in the United States, 2004–2014
Source: BMC Public Health. 2024 Nov 19;24:3211. doi: 10.1186/s12889-024-20669-5 (PMC11575043; doi:10.1186/s12889-024-20669-5)
Supplement: Supplementary file 2 — Supplementary Material 2: Incorrect supplementary file 3 [file 12889_2024_20669_MOESM2_ESM.docx]

**SUPPLEMENT TABLE 3.a Full results from the main logistic regression for the association between AL at MIDUS 2 Biomarker Project and CP status at MIDUS 3**

|  | **OR (95% CI)** | **P-value** |
| --- | --- | --- |
| **Allostatic load pattern** |  |  |
| Baseline | Ref |  |
| Parasympathetic dysregulation | 1.03 (0.67, 1.57) | 0.889 |
| Metabolic dysregulation | 0.85 (0.55, 1.31) | 0.464 |
| **Year gap between data collections** |  |  |
| MIDUS 2 Biomarker Project to MIDUS 3 | 0.88 (0.77, 1.01) | 0.069 |
| **Education** |  |  |
| high school or less | Ref |  |
| bachelor's degree | 1.15 (0.77, 1.7) | 0.497 |
| Master's degree and above | 1.03 (0.65, 1.63) | 0.899 |
| **Age** | 1.01 (0.99, 1.03) | 0.190 |
| **Marital Status** |  |  |
| Married | Ref |  |
| Divorced & Separated | 1.21 (0.74, 1.98) | 0.450 |
| Never married & Widowed | 1.63 (0.96, 2.76) | 0.071 |
| **Income-to-needs ratio** |  |  |
| Affluent | Ref |  |
| Adequate-income | 0.8 (0.54, 1.18) | 0.262 |
| Low-income or below | 0.43 (0.27, 0.71) | 0.001 |
| **Race/ethnicity** |  |  |
| White | Ref |  |
| Non-white | 0.65 (0.34, 1.26) | 0.203 |
| **Gender** |  |  |
| Male | Ref |  |
| Female | 0.73 (0.51, 1.04) | 0.085 |
| **Total number of Metabolic Equivalent of Task (MET) minutes per week** |  |  |
| 500-1000 | Ref |  |
| Greater than 1000 | 0.61 (0.38, 0.97) | 0.036 |
| Less than 500 | 0.94 (0.59, 1.5) | 0.797 |
| **Smoking behavior** |  |  |
| Current Smoker | Ref |  |
| Ex-Smoker | 1.15 (0.65, 2.02) | 0.640 |
| non-Smoker | 1.49 (0.87, 2.54) | 0.145 |
| **Drinking behavior** |  |  |
| Moderate + drinker | Ref |  |
| Light drinker | 0.84 (0.56, 1.27) | 0.408 |
| Non-drinker or rarely drink | 0.88 (0.58, 1.33) | 0.543 |
| **Childhood parent emotional abuse** |  |  |
| 1 (Never) | Ref |  |
| 1.5 | 0.63 (0.37, 1.08) | 0.093 |
| 2 | 0.95 (0.62, 1.46) | 0.826 |
| 2.5 | 1.18 (0.76, 1.81) | 0.461 |
| 3 (Most frequent) | 0.87 (0.59, 1.27) | 0.469 |
| **Childhood parent physical abuse** |  |  |
| 1 (Never) | Ref |  |
| 1.5 | 1.06 (0.57, 1.97) | 0.863 |
| 2 | 1.34 (0.83, 2.16) | 0.230 |
| 2.5 | 0.99 (0.62, 1.58) | 0.955 |
| 3 (Most frequent) | 1.45 (0.97, 2.15) | 0.068 |
| **Medication intake** |  |  |
| Yes | Ref |  |
| No | 0.48 (0.31, 0.73) | 0.001 |
| **Multimorbidity** |  |  |
| <2 | Ref |  |
| 2+ | 0.64 (0.39, 1.02) | 0.062 |

**SUPPLEMENT TABLE 3.b Full results from the main multinomial logistic regression for the association between AL at MIDUS 2 Biomarker Project and CP interference and the number of CP sites at MIDUS 3**

|  | **CP interference** | | | | **The number of pain locations** | | | |
| --- | --- | --- | --- | --- | --- | --- | --- | --- |
| **No pain vs** | **Low interference pain** | | **High interference pain** | | **1-2 pain locations** | | **3+ pain locations** | |
|  | **RRR (95% CI)** | **P-value** | **RRR (95% CI)** | **P-value** | **RRR (95% CI)** | **P-value** | **RRR (95% CI)** | **P-value** |
| **Allostatic load pattern** |  |  |  |  |  |  |  |  |
| Baseline | Ref |  | Ref |  | Ref |  | Ref |  |
| Parasympathetic dysregulation | 0.87 (0.54, 1.39) | 0.552 | 1.24 (0.65, 2.39) | 0.512 | 0.84 (0.51, 1.36) | 0.474 | 1.30 (0.69, 2.44) | 0.411 |
| Metabolic dysregulation | 0.92 (0.56, 1.52) | 0.742 | 2.00 (1.06, 3.79) | 0.033 | 0.89 (0.54, 1.47) | 0.654 | 2.03 (1.08, 3.83) | 0.029 |
| **Year gap between data collections** |  |  |  |  |  |  |  |  |
| MIDUS 2 Biomarker Project to MIDUS 3 | 1.13 (0.97, 1.32) | 0.107 | 1.13 (0.92, 1.39) | 0.239 | 1.14 (0.98, 1.33) | 0.094 | 1.13 (0.93, 1.39) | 0.224 |
| **Education** |  |  |  |  |  |  |  |  |
| high school or less | Ref |  | Ref |  | Ref |  | Ref |  |
| bachelor's degree | 0.86 (0.56, 1.34) | 0.518 | 0.87 (0.47, 1.61) | 0.653 | 1.04 (0.67, 1.62) | 0.858 | 0.6 (0.32, 1.12) | 0.108 |
| Master's degree and above | 0.82 (0.48, 1.39) | 0.464 | 1.31 (0.66, 2.59) | 0.436 | 1.03 (0.61, 1.73) | 0.911 | 0.9 (0.45, 1.81) | 0.770 |
| **Age** | 0.99 (0.97, 1.01) | 0.466 | 0.98 (0.96, 1.01) | 0.158 | 0.98 (0.97, 1) | 0.114 | 1 (0.97, 1.02) | 0.842 |
| **Marital Status** |  |  |  |  |  |  |  |  |
| Married | Ref |  | Ref |  | Ref |  | Ref |  |
| Divorced & Separated | 0.92 (0.53, 1.6) | 0.768 | 0.65 (0.3, 1.39) | 0.267 | 0.83 (0.47, 1.47) | 0.519 | 0.75 (0.37, 1.54) | 0.440 |
| Never married & Widowed | 0.74 (0.41, 1.33) | 0.319 | 0.37 (0.15, 0.94) | 0.037 | 0.59 (0.32, 1.1) | 0.099 | 0.58 (0.26, 1.3) | 0.189 |
| **Income-to-needs ratio** |  |  |  |  |  |  |  |  |
| Affluent | Ref |  | Ref |  | Ref |  | Ref |  |
| Adequate-income | 1.28 (0.82, 1.99) | 0.281 | 1.17 (0.65, 2.11) | 0.602 | 1.26 (0.8, 1.96) | 0.319 | 1.24 (0.69, 2.22) | 0.466 |
| Low-income or below | 2.55 (1.5, 4.34) | 0.001 | 1.82 (0.87, 3.83) | 0.114 | 2.1 (1.21, 3.66) | 0.009 | 2.63 (1.32, 5.25) | 0.006 |
| **Race/ethnicity** |  |  |  |  |  |  |  |  |
| White | Ref |  | Ref |  | Ref |  | Ref |  |
| Non-white | 1.6 (0.78, 3.31) | 0.203 | 1.41 (0.52, 3.8) | 0.495 | 1.24 (0.57, 2.71) | 0.583 | 2.47 (1.02, 5.99) | 0.046 |
| **Gender** |  |  |  |  |  |  |  |  |
| Male | Ref |  | Ref |  | Ref |  | Ref |  |
| Female | 1.06 (0.71, 1.58) | 0.780 | 2.46 (1.39, 4.36) | 0.002 | 0.93 (0.62, 1.39) | 0.720 | 3.34 (1.86, 5.98) | 0.000 |
| **Total number of Metabolic Equivalent of Task (MET) minutes per week** |  |  |  |  |  |  |  |  |
| 500-1000 | Ref |  | Ref |  | Ref |  | Ref |  |
| Greater than 1000 | 1.58 (0.94, 2.65) | 0.084 | 1.83 (0.88, 3.77) | 0.104 | 1.54 (0.9, 2.61) | 0.114 | 1.82 (0.91, 3.6) | 0.088 |
| Less than 500 | 1.05 (0.61, 1.78) | 0.869 | 1.14 (0.56, 2.34) | 0.722 | 1.17 (0.68, 2.01) | 0.561 | 0.86 (0.43, 1.72) | 0.662 |
| **Smoking behavior** |  |  |  |  |  |  |  |  |
| Current Smoker | Ref |  | Ref |  | Ref |  | Ref |  |
| Ex-Smoker | 1.11 (0.57, 2.17) | 0.759 | 0.57 (0.26, 1.24) | 0.154 | 1.1 (0.55, 2.18) | 0.788 | 0.61 (0.29, 1.3) | 0.202 |
| non-Smoker | 1 (0.53, 1.87) | 0.994 | 0.31 (0.15, 0.65) | 0.002 | 0.98 (0.51, 1.87) | 0.945 | 0.34 (0.16, 0.71) | 0.004 |
| **Drinking behavior** |  |  |  |  |  |  |  |  |
| Moderate + drinker | Ref |  | Ref |  | Ref |  | Ref |  |
| Light drinker | 1.17 (0.75, 1.85) | 0.490 | 1.27 (0.66, 2.44) | 0.473 | 1.21 (0.76, 1.95) | 0.418 | 1.13 (0.62, 2.08) | 0.683 |
| Non-drinker or rarely drink | 0.9 (0.56, 1.44) | 0.649 | 1.81 (0.97, 3.37) | 0.061 | 1.23 (0.77, 1.97) | 0.382 | 0.97 (0.52, 1.8) | 0.913 |
| **Childhood parent emotional abuse** |  |  |  |  |  |  |  |  |
| 1 (Never) | Ref |  | Ref |  | Ref |  | Ref |  |
| 1.5 | 1.45 (0.79, 2.66) | 0.236 | 1.9 (0.85, 4.24) | 0.116 | 1.65 (0.9, 3.03) | 0.106 | 1.29 (0.57, 2.91) | 0.539 |
| 2 | 0.95 (0.59, 1.55) | 0.851 | 1.35 (0.72, 2.55) | 0.348 | 0.96 (0.59, 1.57) | 0.877 | 1.23 (0.67, 2.26) | 0.504 |
| 2.5 | 1.1 (0.67, 1.79) | 0.716 | 0.44 (0.21, 0.92) | 0.029 | 0.82 (0.51, 1.32) | 0.406 | 0.92 (0.45, 1.86) | 0.811 |
| 3 (Most frequent) | 1.2 (0.78, 1.83) | 0.407 | 1.26 (0.66, 2.4) | 0.489 | 0.97 (0.64, 1.48) | 0.898 | 1.73 (0.93, 3.2) | 0.082 |
| **Childhood parent physical abuse** |  |  |  |  |  |  |  |  |
| 1 (Never) | Ref |  | Ref |  | Ref |  | Ref |  |
| 1.5 | 0.83 (0.41, 1.7) | 0.616 | 1.25 (0.51, 3.09) | 0.629 | 0.66 (0.31, 1.39) | 0.272 | 1.73 (0.72, 4.21) | 0.223 |
| 2 | 0.83 (0.48, 1.44) | 0.509 | 0.67 (0.33, 1.34) | 0.252 | 0.64 (0.35, 1.15) | 0.134 | 0.9 (0.47, 1.73) | 0.757 |
| 2.5 | 1.25 (0.73, 2.16) | 0.414 | 0.66 (0.33, 1.31) | 0.235 | 1.01 (0.59, 1.75) | 0.962 | 0.86 (0.44, 1.69) | 0.656 |
| 3 (Most frequent) | 0.73 (0.46, 1.16) | 0.184 | 0.71 (0.4, 1.26) | 0.238 | 0.65 (0.42, 1.02) | 0.064 | 0.8 (0.45, 1.43) | 0.451 |
| **Medication intake** |  |  |  |  |  |  |  |  |
| Yes | Ref |  | Ref |  | Ref |  | Ref |  |
| No | 2.01 (1.24, 3.26) | 0.005 | 2.42 (1.18, 4.99) | 0.016 | 2.01 (1.23, 3.29) | 0.005 | 2.53 (1.25, 5.11) | 0.010 |
| **Multimorbidity** |  |  |  |  |  |  |  |  |
| <2 | Ref |  | Ref |  | Ref |  | Ref |  |
| 2+ | 1.51 (0.89, 2.56) | 0.131 | 1.8 (0.79, 4.1) | 0.162 | 1.48 (0.87, 2.51) | 0.151 | 1.97 (0.87, 4.47) | 0.105 |
